# Management of Acute Lymphoblastic Leukemia During Pregnancy: A Case Report and Review of the Literature

**DOI:** 10.7759/cureus.52489

**Published:** 2024-01-18

**Authors:** Saleha Nadeem, Ehsan Elahi, Imran Iftikhar, Sobia Umar, Bushra Ahsan, Usman Ahmad, Syed W Bokhari

**Affiliations:** 1 Medical Oncology, Shaukat Khanum Memorial Cancer Hospital and Research Centre, Lahore, PAK; 2 Medical Onocology, Shaukat Khanum Memorial Cancer Hospital and Research Centre, Lahore, PAK

**Keywords:** pegasperginase, intrathecal cytarabine, intrathecal methotrexate, pregnancy, acute lymphoblastic leukemia

## Abstract

Acute lymphoblastic leukemia (ALL) during pregnancy necessitates treatment with high-dose chemotherapy, which can threaten the lives of both the mother and fetus. The aim of the treatment not only focuses on selecting and administering optimal chemotherapy with appropriate doses to the mother but also reflects the crucial understanding of the fetal gestational age at the time of administration of chemotherapy to minimize fetal exposure. We describe the case of a 19-year-old patient diagnosed with ALL at 29 weeks gestation. She received treatment in the third trimester with the Berlin-Frankfurt-Munster (BFM) 2000 induction chemotherapy protocol consisting of a combination of daunorubicin, vincristine, pegaspargase, prednisolone, and intrathecal (IT) methotrexate and gave birth to a healthy baby girl via vaginal delivery four weeks after initiating the induction of chemotherapy.

## Introduction

A cancer diagnosis during pregnancy is hurtful and presents a significant challenge for the patient, her family, and the medical staff. Recent data from the largest health database in the United States reports an increase in the likelihood of cancer associated with pregnancy from 3.41 in 2004 to 4.33 in 2013 per 10,000 deliveries [1]. Among hematologic malignancies, Hodgkin’s lymphoma is the most prevalent cancer in pregnant women, whereas leukemias are comparatively uncommon types encountered during pregnancy. Since leukemias are so rare, this has impeded large prospective controlled trials; hence, evidence is restricted to retrospective reports and case series, which gives rise to difficulties in developing guidelines addressing their management during pregnancy [2-4].

In everyday clinical practice, the diagnosis of malignancy during pregnancy is challenging and requires making many difficult decisions, taking into account several ethical, personal, and clinical factors. Early diagnosis guarantees the optimal outcome of most malignancies. However, due to the overlapping signs and symptoms of many hematological malignancies and pregnancy, combined with the avoidance of invasive and radiological diagnostic procedures during gestation, there is often a delay in diagnosis and subsequent management of the patients. Administration of chemotherapy in the first trimester of pregnancy is likely to be lethal to the fetus and poses an increased risk of fetal growth retardation and congenital malformations.

The most recent evidence suggests that cautious administration of the majority of chemotherapeutic agents to pregnant women in the second and third trimesters has no significant adverse impact on the growth and health of the baby and that most pregnant women should wait for term and aim for a vaginal birth [5,6]. In this report, we present our patient diagnosed with acute lymphoblastic leukemia (ALL) and her treatment with the Berlin-Frankfurt-Munster (BFM) 2000 protocol [7] during pregnancy, along with a concise review of the literature on the treatment of pregnancy-associated ALL and the long-term outcomes of children exposed to chemotherapy in utero.

## Case presentation

**Figure 1:**
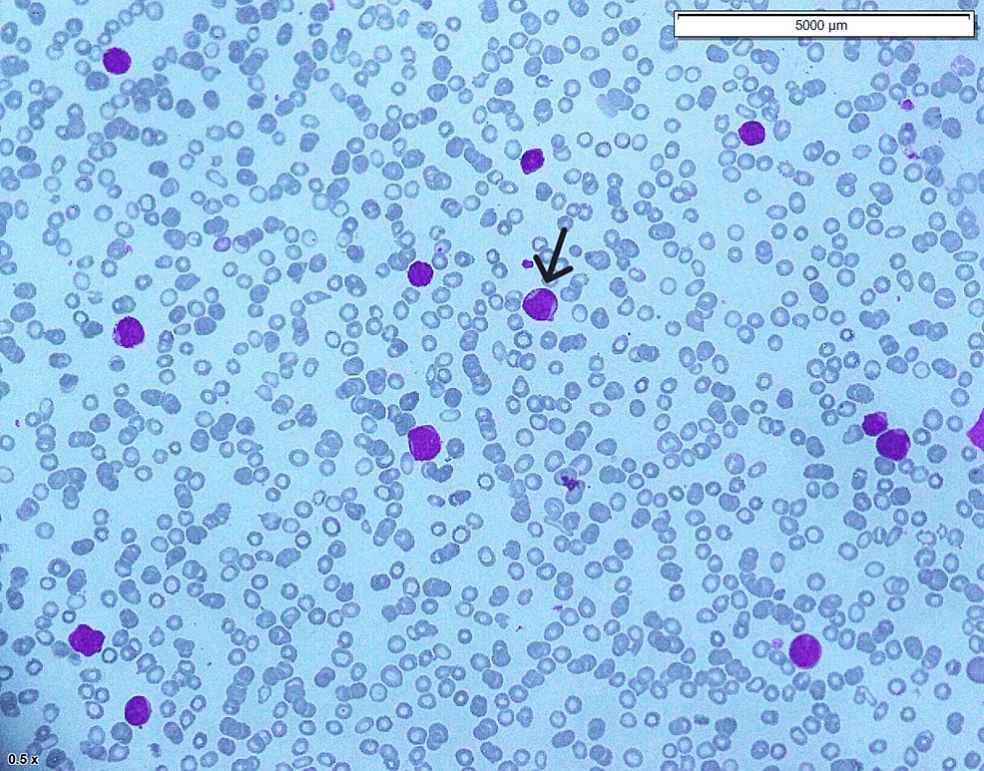
Bone marrow aspirate The bone marrow aspirate shows small to medium-sized blasts (black arrow) with open chromatin, high nuclear-to-cytoplasmic ratio, and scant cytoplasm. No granules or Auer rods are seen.

**Figure 2:**
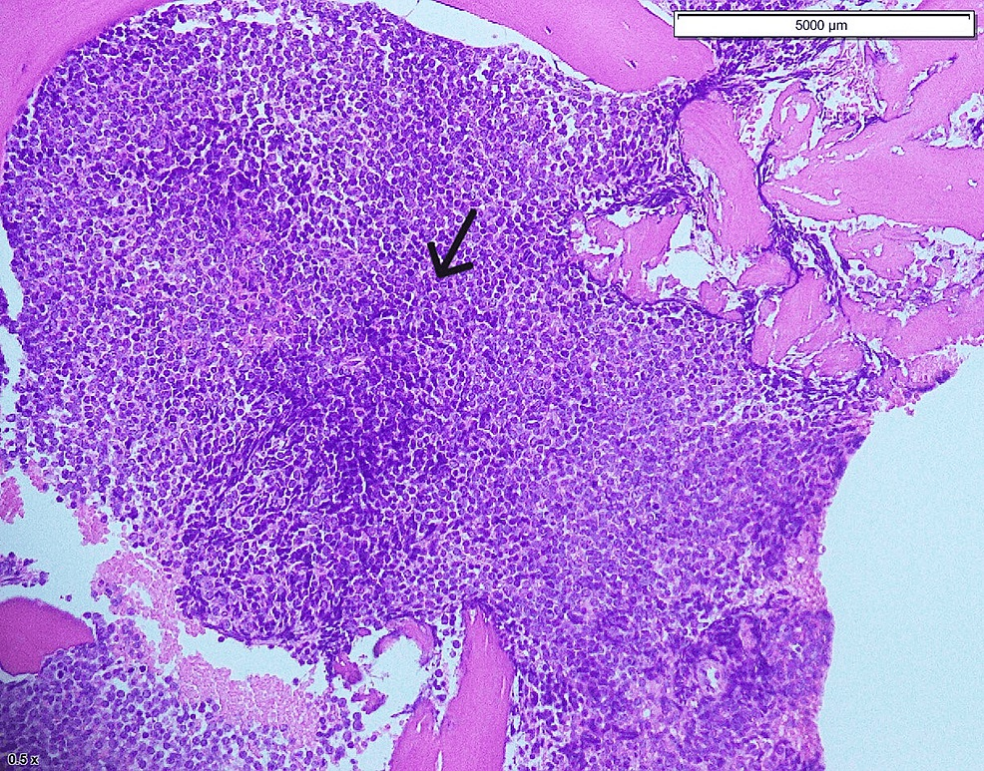
Bone marrow trephine The bone marrow trephine shows that 90% of the bone marrow is infiltrated by blast cells (black arrow).

**Figure 3:**
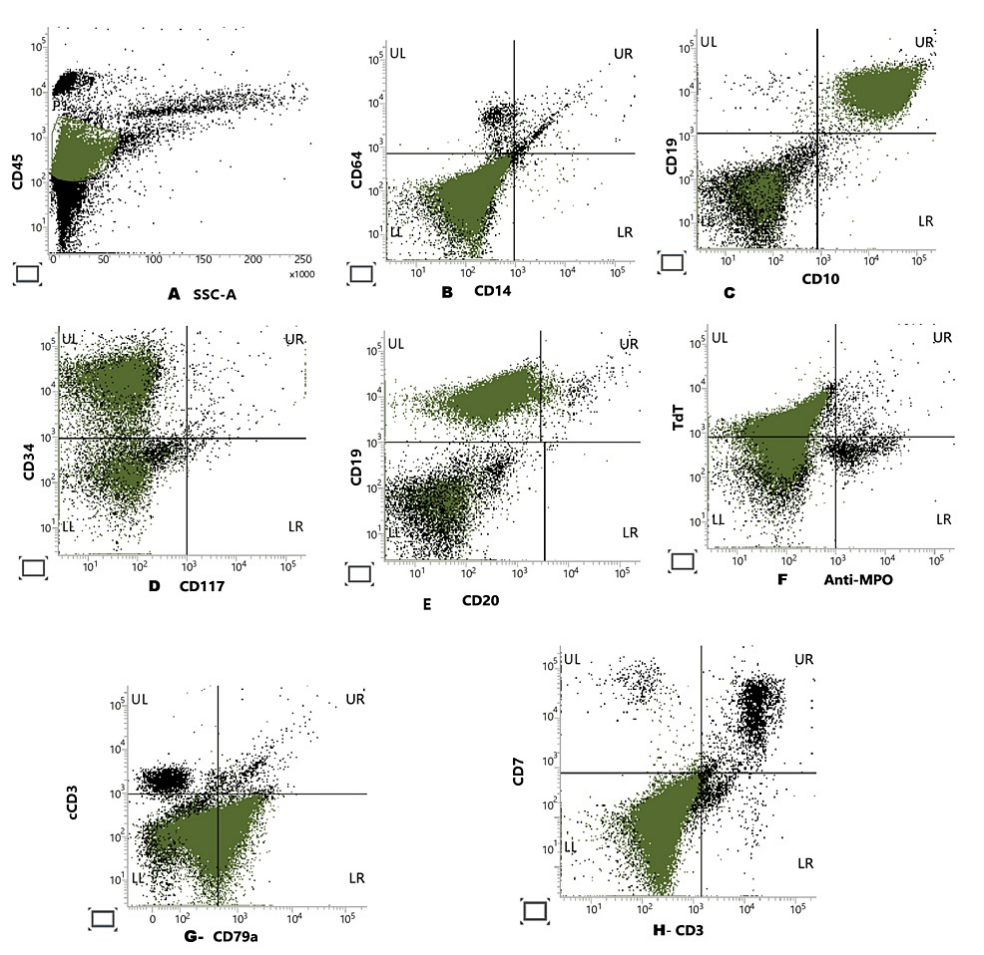
Acute leukemia immunophenotyping by flow cytometry A: CD45 dim positive, B: CD14 and CD64 negative, C: CD19 and CD10 positive, D: CD34 positive and CD117 negative, E: CD19 positive and CD20 negative, F: TDT positive and MPO negative, G: CD3 negative and CD79a positive, H: CD3 and CD7 negative

A 19-year-old pregnant female at 29 weeks gestation with no family history of cancer presented to the walk-in clinic with a one-month history of sore throat, fever, myalgia, backache, fatigue, and shortness of breath, along with gum bleeding. Initial baseline laboratory investigations revealed pancytopenia with a white blood cell count of 1.06 ×103/µl, hemoglobin 7.9 g/dL, platelets 40×103/μl, and an absolute neutrophil count (ANC) of 0.08×103/μl (Table 1). She was referred to the emergency assessment room for an urgent workup. A bone marrow aspirate and biopsy were performed, which confirmed precursor B-cell acute lymphoblastic leukemia (Pre-B ALL), comprising 71% blasts (Figures 1-2). Flow cytometry revealed these blasts to be expressing HLA-DR++, CD45 dim+, CD34++, CD19+, CD10++, TDT+, and CD79a dim+ and were negative for MPO, CD3, CD20, and all other antigens (Figure 3).

**Table 1:** Laboratory data APTT: Activated partial thromboplastin time, PT: Prothrombin time, INR: International normalized ratio, HBsAg: Hepatitis B surface antigen, HCV: Hepatitis C virus

Variables	Reference range		Day 1	Day 22	Day 27
Hematology					
Blood counts (x10^3^/µL)	4-10		1.06	2.7	4.54
Hemoglobin (g/dL)	12-15		7.9	8.7	9.2
Hematocrit (%)	36-46		19.4	26.2	28
Platelets(x10^3^/µL)	150-450		40	55	155
Lymphocytes(x10^3^/µL)	1-5		0.67	1.04	1.13
Neutrophils (x10^3^/µL)	2-8		0.05	1.6	3.32
Coagulation					
APTT (secs)	2-31.9		22.0	-	-
PT (secs)	9.9-11.8		9.3	-	-
INR	0.9–1.1		0.87	-	-
Serology				-	-
HBsAg	-		Negative	-	-
HCV	-		Negative	-	-
HIV I & II	-		Negative	-	-

Cytogenetics revealed a normal female karyotype 46 XX. Neither the Philadelphia chromosome (BCR-ABL translocation or rearrangement) by fluorescent in situ hybridization (FISH) nor the BCR-ABL breakpoint fusion by reverse transcriptase polymerase chain reaction (RT-PCR) were identified. The FISH for the MLL gene rearrangement was negative.

Ultrasound abdomen and pelvis revealed a single alive intrauterine fetus of gestational age of 29 weeks with good cardiac activity and fetal movements. Normal amniotic fluid with no obvious fetal anomaly was reported. Meanwhile, treatment for ALL was started initially with oral prednisolone 60 mg/m2/day along with allopurinol once daily. The patient was referred for an urgent obstetrics consultation. The obstetrician advised a weekly ultrasound scan and planned to deliver the baby at around 34+ weeks of pregnancy, preferably via vaginal delivery, with a Caesarean section to be considered only on any obstetric indication with adequate platelet counts.

Our leukemia multidisciplinary team meeting meanwhile recommended induction chemotherapy with the BFM 2000 protocol intraarterial (IA) containing methotrexate 12 mg intrathecal (IT) for days 1, 12, and 33; vincristine 1.5 mg/m2 on days 8, 15, 22, and 29; daunorubicin 30 mg/m2 on days 8, 15, 22, and 29; pegaspargase 1000 mg/m2 on days 12, and 24; and prednisolone 60 mg/m2 once a day on days 1 to 28; and then taper and cease (Table 2). To prevent fetal complications, it was decided to avoid pegaspargase and to replace IT methotrexate with IT cytarabine prior to delivery. For initial prophylaxis, only acyclovir and ciprofloxacin were given, whereas co-trimoxazole and azole antifungals were held until successful delivery. The aim was to maintain platelets and ANC at >20×103/μl and >1.0×103/μl, respectively, with granulocyte-colony stimulating factor (GCSF) and transfusion support.

**Table 2:** BFM 2000 protocol with modifications in induction IA protocol BFM: Berlin-Frankfurt-Munster, IA: Intraarterial, IT: Intrathecal, ANC: Absolute neutrophils count, BMA: Bone marrow aspirate, MRD: Minimal residual disease

Phase	Planned chemotherapy	Adjustments for pregnancy	Actual administration	Outcome
Induction (days 1 to 33)	Methotrexate 12 mg IT (days 1, 12, 33)	Methotrexate replaced with cytarabine IT for pre-delivery	Days 1 to 21 administered as planned	ANC and platelets recovered well after delivery.
	Vincristine 1.5 mg/m2 (days 8, 15, 22, 29)	Acyclovir and ciprofloxacin for initial prophylaxis	Days 22 & 29 were held due to planned delivery at 35 weeks.	Day 33 BMA: remission marrow, MRD positive (1.9%) for pre-B-ALL
	Daunorubicin 30 mg/m2 (days 8, 15, 22, 29)	Co-trimoxazole and azole antifungals were held until delivery.	Day 22 administered postpartum (day 4)	-
	Pegaspargase 1000 mg/m2 (Days 12, 24)	Pegaspargase was omitted due to fetal concerns	Day 24 administered postpartum (day 6)	Day 29 was omitted due to a one-week delay
	Prednisolone 60 mg/m2 daily (days 1 to 28) then taper and cease	-	-	-
Consolidation protocol IB	Cyclophosphamide IV 1000 mg/m2 (days 1 and 29), mercaptopurine PO 60 mg/m2 (days 1 to 28), cytarabine S/C 75 mg/m2 (days 3 to 6, 10 to 13, 17 to 20 and 24 to 27), IT methotrexate 12 mg (says10 and 24)	-	Administered as planned	BMA MRD negative after consolidation
Protocol M	Mercaptopurine 25 mg/m2 (days 1 to 56), methotrexate 500 mg/m2 (days 8, 22, 36, 50), high dose ,ethotrexate 4500 mg/m2 (days 8, 22, 36, 50), IT methotrexate 12 mg (days 8, 22, 36, 50)	-	Administered as planned	BMA MRD borderline positive (0.03%) after protocol M
Reinduction	Dexamethasone PO 10 mg/m2 (days 1 to 21, then taper and cease), doxorubicin IV 30 mg/m2 (days 8, 15, 22, 29), vincristine IV 1.5 mg/m2 (days 8, 15, 22, 29), L-asparaginase IV 10,000 IU/m2 (days 8, 11, 15, 18), thioguanine PO 60 mg/m2 (days 36 to 49), cyclophosphamide IV 1000 mg/m2 (day 36), cytarabine S/C 75 mg/m2 (days 38 to 41 and 45 to 48), IT methotrexate 12 mg (days 38 and 45)	-	Administered as planned	Post-re-induction BMA: inadequate sample for morphology, MRD borderline positive (0.03%)
Maintenance phase	Mercaptopurine PO 50 mg/m2 titrated once daily(24 months), methotrexate PO 20 mg/m2 titrated once weekly (24 months)	Currently on protocol M maintenance therapy	Administered as planned	Ongoing

The patient was safely administered the planned induction chemotherapy until day 21; on days 22 and 29, chemotherapy was held as the patient was planned for admission under obstetrics to proceed with the delivery of the baby in the upcoming week. This allowed her full blood counts to recover with an ANC of 1.61×103/μl and a platelet count of 55×103/μl. At 35 weeks of gestation and four weeks after the initiation of BFM 2000 induction chemotherapy, she gave birth to a baby girl via vaginal delivery. Her postpartum full blood count showed an ANC of 3.3×103/μl and a platelet count of 155×103/μl.

On postpartum days 4 and 6, i.e., day 22 and day 24, respectively, the patient was re-initiated on her interrupted chemotherapy (withheld due to delivery); she was administered chemotherapy (vincristine and daunorubicin) on day 22 and pegaspargase on day 24. Prophylactic co-trimoxazole and azole antifungals were commenced as well. Due to the one-week interruption and delay in protocol to allow for safe delivery, day 29's chemotherapy was omitted, and the patient was discharged. A bone marrow aspirate (BMA) for the assessment of minimal residual disease (MRD) by flow cytometry was performed on day 33 (end of induction therapy), which showed remission marrow with no blast cells by morphology but positive MRD for pre-B-ALL at 1.9%.

The patient was started on the BFM 2000 consolidation protocol IB chemotherapy, following which her BMA and MRD came back negative. She was then commenced on protocol M. Bone marrow aspirate and MRD for pre-B-cell ALL remained negative after consolidation and protocol M chemotherapy. She finished the BFM 2000 re-induction protocol II and started maintenance therapy. Post-re-induction BMA was identified as an inadequate sample for morphology, but MRD came out to be borderline positive at 0.03%. A repeat BMA and MRD have been booked. At the time of writing this report, the patient has been started on maintenance therapy. Her baby girl continues to do well and has reached normal growth at seven months of age.

## Discussion

Among hematological disorders, ALL is a rare cancer encountered during pregnancy. Approximately 1 in 75,000 to 100,000 pregnancies is affected by acute leukemia, and only one-third of this incidence represents ALL, whereas the remaining two-thirds represent acute myeloid leukemia (AML) [8]. Due to the scarcity of data related to the association of ALL with pregnancy, all relevant literature is in the form of case reports or case series. Table 3 summarizes the data in the form of case reports and case series reporting about ALL in pregnancy. Vincristine, daunorubicin, and prednisolone were used as backbone therapy for ALL treatments during pregnancy. Delay in the initiation of induction chemotherapy may result in poor maternal outcomes, but a delay for a shorter duration for delivery first may be reasonable in females who present in the late phase of the third trimester. In most of the data reported, if ALL was diagnosed during the first trimester of pregnancy, then pregnancy was terminated due to the risk of teratogenic effects of chemotherapeutic agents on the fetus [9-11]. If ALL was diagnosed during the late phase of the third trimester, then delivery was a priority instead of initiating chemotherapy [12-13]. Patients who present during the second or early third trimester are more challenging, and administration of multi-agent chemotherapy is necessary because waiting for the patient to complete gestation time may affect maternal outcomes [14-19].

**Table 3:** Case reports of ALL managed during pregnancy *Pre-T ALL; ^a^Patient had leukemia in remission and was on maintenance therapy 6-MP and intravenous MTX at the time of conception and continued maintenance until relapsed at week 21; ^b^Patient had leukemia in remission and after 11 months of completion of maintenance chemotherapy and at week 32 of gestation she was diagnosed with relapsed ALL; ^c^Data of gestational age in weeks at the time of delivery not available. ALL: Acute lymphoblastic leukemia; Allo-SCT: Allogenic stem cell transplant Amsac: Amsacrine; AraC: Cytarabine; CHF: Congestive heart failure; CR: Complete Remission; CR 1: First complete remission; CR 2: Second complete remission; Cyclo: Cyclophosphamide; Dexa: Dexamethasone; DNR: Daunorubicin; DXR: Doxorubicin; GA: Gestational age; HCT: Hydrocortisone; Ida: Idarubicin; IT: Intrathecal; IVD: Induced vaginal delivery; L-Asp: L-Asparaginase; MTX: Methotrexate; NR: Not reported; Pred: Prednisolone; SVD: Spontaneous vaginal delivery; Tg: Thioguanine; VCR: Vincristine; 6-MP: 6-Mercaptopurine

Patient	Study	Age at diagnosis	GA at therapy (in weeks)	Induction therapy during pregnancy	Pregnancy and fetal outcome	Response to therapy and patient outcome (as per study)
1	Molkenboer et al. [9]	30	6	Pred, VCR, L-Asp, DNR, IT MTX	Missed abortion at 11 weeks	CR, followed by Allo-SCT which was complicated by sepsis and died
2		37	15	Pred, VCR, L-Asp, DNR, IT MTX, AraC	Spontaneous delivery of stillborn fetus at week 22	Unable to achieve remission and died
3	Aljurf et al. [10]	25	Induction chemotherapy started after delivery	VCR, Dexa, Ida, L-Asp	14 weeks GA at diagnosis, spontaneous abortion at 14 weeks	Short remission followed by recurrence of refractory leukemia and death three months later
4		37	29	VCR, Dexa, Ida, L-Asp	Full term^c^ anemic infant with vaginal delivery	CR after induction but relapsed at three months and died
5		21*	Induction chemotherapy started after delivery	VCR, Dexa, Ida, L-Asp	30 weeks GA at diagnosis, anemic infant with vaginal delivery at 30 weeks	CR with induction. Relapsed at six months followed by allo-SCT in CR 2. Relapsed again at 10 months post SCT. Expired.
6		41	Induction chemotherapy started after delivery	Cyclo, VCR, Dexa, Ida, L-Asp	11 weeks GA at diagnosis, therapeutic abortion at 11 weeks	CR post-induction. Relapsed after two months followed by allo-SCT in CR 2. Relapsed again after three years of CR. Expired.
7		27	13	NR	During induction had spontaneous abortion at week 14	CR 1. Alive four years later.
8	Farhadfar et al. [11]	26	Induction chemotherapy started after delivery	VCR, DXR, L-Asp, Pred, Cyclo	12 weeks GA at diagnosis, elective termination at week 14	Disease outcome not reported, alive
9		23	Induction chemotherapy started after delivery	VCR, DXR, L-Asp, Pred, Cyclo	Six weeks GA at diagnosis, elective termination at week 7	CR 1, delivered a healthy baby 43 months post-diagnosis, alive
10		34	10	CALGB9111 protocol: VCR, DNR, L-Asp, Pred, Cyclo	Spontaneous abortion at 22 weeks	CR 2, Allo-SCT complicated by septic shock and died
11		19	16	VCR, DNR, Pred	Fetal loss by stillbirth at 22 weeks	CR after induction, died of respiratory failure
12		28	Induction chemotherapy started after delivery	DNR, VCR, Pred	35 weeks GA at diagnosis, IVD at 38 weeks, healthy baby	CR after induction, relapsed 15 months after diagnosis but died of refractory ALL
13	Terek et al. [12]	21	Induction chemotherapy started after delivery	VCR, DNR, Pred, L-Asp	31 weeks GA at diagnosis, caesarean section at 32 weeks, newborn with respiratory distress required intubation	Maternal death due to sepsis
14	Vlijm-Kievit et al. [13]	37*	Induction chemotherapy started after delivery	Only Pred (prephase) administered until delivery. VCR, DNR, L-Asp, Pred	36 weeks GA at diagnosis, IVD at weeks 37. Healthy infant	CR, alive
15	Hansen et al. [14]	24	26	CALGB 911: Cyclo, DNR, VCR, Pred, L-Asp (Course 1 Induction) IT MTX, Cyclo, 6-MP, AraC, VCR, L-Asp (Course 2a & 2b Intensification)	Spontaneous vaginal delivery at 36 weeks. Healthy infant	NR
16	Papantoniou et al. [15]	16	26	BFM 95 protocol: DNR, VCR, Pred, L-Asp, IT MTX	Caesarean section at 32 weeks. Baby with normal growth at 18 months.	Remission. On maintenance at 18 months, alive
17	Matsouka et al. [16]	16	26	BFM 95 protocol: DNR, VCR, Pred, L-Asp, IT MTX	Caesarean section at 33 weeks. Healthy infant	CR, alive
18	Udink Ten Cate et al. [17]	30	23	DNR, VCR, Cyclo, Pred (1^st^ course) Cyclo, AraC, Tg, IT MTX (2^nd^ course) AraC, Amsac (second line)	Spontaneous vaginal delivery at 33 weeks, pancytopenic infant. Normal development at 24-month follow-up.	Matched unrelated donor Allo-SCT. In CR at two years after transplantation, alive
19	Khandaker et al. [18]	25	24	Pred, IT MTX, VCR, L-Asp (induction) VCR, IT MTX, 6-MP (maintenance)	Caesarean section at 34 weeks. Normal infant	Remission, alive
20	Mainor et al. [19]	38	24	HyperCVAD protocol: Cyclo, Dexa, VCR, DXR, IT AraC (same cycle repeated as second cycle)	Caesarean section at 30 weeks. Healthy baby with normal growth at three months	Post cycle three of chemotherapy became septic and died.
21	O’Donnell et al. [20]	24	15	TAD regimen (Tg, AraC, DNR)	Intrauterine fetal death at 30 weeks due to pre-eclampsia	CR, alive
22	Okun et al. [21]	18	12	VCR, Pred, IT MTX (1^st^ induction) Cyclo, L-Asp, DNR (2^nd^ induction)	Caesarean section at 31 weeks, newborn with transient pancytopenia, CHF, but normal development after one year	CNS relapses at five weeks after delivery. Remained in remission 20 months after diagnosis. Died of sepsis.
23	Dara et al. [22]	26^a^	21	DXR, VCR, Pred (re-induction chemotherapy for relapse ALL) AraC, MTX (maintenance)	Caesarean section at 36 weeks. Baby born with polycythemia and hyperbilirubinemia. Normal growth at six months	Remission, alive
24	Tewari et al. [23]	17^b^	33	VCR, Pred (re-induction chemotherapy for relapse ALL)	IVD at 35 weeks gestation, healthy infant	Consolidation therapy followed by transplant, 22 months of remission post-transplant, alive
25	Camera et al. [24]	21	17	VCR, DNR, L-Asp, Pred	Caesarean section at 29 weeks. Healthy infant	Refractory leukemia so died nine months later
26	Zaidi et al. [25]	16	12	DNR, VCR, L-Asp, Pred, IT MTX	Spontaneous complete abortion at 16 weeks	Remission after induction, but died four weeks after induction due to coagulopathy
27		17	21	COG A5971 protocol (minor modification for pregnancy): Pred, DNR, VCR, IT AraC, IT HCT (Induction) Cyclo, AraC, 6-MP	Caesarean section at 32 weeks. Infant with normal growth at 17 days.	CR, receiving maintenance, alive
28	Zhu et al. [26]	19	4	Declined therapy	Fetal loss	Mother died shortly after discharge from hospital
29		33	34	Cyclo, DNR, VCR, Pred	IVD^c^ at full-term healthy baby	CR, died of pulmonary infection eight months after diagnosis
30		41	27	Cyclo, DNR, VCR, Pred	Caesarean section^c^, healthy baby	CR, died of refractory ALL at 16 months
31	Retnoningrum et al. [27]	36	27	VCR, Pred	Caesarean section at 32 weeks, healthy infant	NR, patient lost to follow-up

Cancer during pregnancy is a challenging scenario. Acute lymphoblastic leukemia is not a common type of cancer encountered during pregnancy. In the past three decades, oncologists have treated cancer in pregnant patients with combination chemotherapy. This has resulted in positive results for both the mother and the unborn child, especially during the second and third trimesters. Compared to the treatment of a solid tumor or lymphoma during pregnancy, the treatment of ALL poses a greater challenge as it involves the utilization of intensive chemotherapy during the induction phase to achieve remission.

The patient in this case report faced a great challenge of leukemia during pregnancy. She presented with severe pancytopenia along with bleeding symptoms when she first arrived at 29 weeks of pregnancy. Supportive treatment with blood transfusions and symptomatic treatment with antipyretics and antibiotics would neither be curative nor sufficient to ensure a safe pregnancy over a long period of time. In addition, any intervention for delivering the baby via caesarean section could be associated with a high risk of bleeding and infections, as well as potentially life-threatening consequences for both the mother and the baby. Therefore, according to the decision of the obstetrician, we decided to administer induction chemotherapy with the BFM 2000 protocol (a combination of four drugs for high-risk ALL) after vaginal delivery of the baby at 34+ weeks of gestation. This not only provided more time for the fetus to reach maturity but also allowed the patient's marrow to go into morphological remission in time for delivery after the first induction of chemotherapy.

Since anthracyclines, vincristine, and steroids were frequently used during pregnancy, mostly to treat breast cancer and lymphoma, and because this combination has also proven to be effective and successful in treating adult ALL, we chose the BFM 2000 induction protocol for treatment. On the contrary, the use of these drugs at levels that were three times higher than those used to treat lymphoma was also concerning.

Pegaspargase was avoided in this case as pregnancy itself is a pro-thrombotic state and because of its potential life-threatening side effects such as acute pancreatitis, hepatotoxicity, hypofibrinogenemia, and thrombosis. Furthermore, we decided to switch IT methotrexate to IT cytarabine until delivery. Interruption of chemotherapy after day 21 for one week and administration of GCSF were measures taken to lessen the risk of pancytopenia and provide sufficient time for the hematological recovery of both mother and infant. At the time of delivery, the aim is for the leukemia of the patient to be in morphological remission with an improvement in the full blood count, making delivery safer.

A variety of other ALL remission induction regimens have also been used. Other than the four drug regimens (steroids, vincristine, anthracycline, and pegaspargase) we used for our patient, a three-drug combination (steroids, vincristine, and asparaginase) for standard-risk ALL and a hyper-cyclophosphamide, vincristine, adriamycin, and dexamethasone (CVAD) combination have also been used [28-31]. As no BCR-ABL translocation or rearrangement was identified in our patient, the addition of a tyrosine kinase inhibitor (TKI) was not required.

In summary, malignancies in pregnancy represent a challenging scenario requiring the consideration of gestational status, type of malignancy, therapeutic options, and their doses by the oncologist to achieve optimal outcomes for the mother and the infant. Based on our case report and literature review, it is recommended that patients with ALL presenting in the first trimester of pregnancy be referred for termination of pregnancy followed by standard chemotherapy for ALL. For those presenting with ALL in the late third trimester of pregnancy, delivery of the baby should be done prior to commencing chemotherapy (although steroids can be safely started). For patients presenting with ALL in the second or early third trimester of pregnancy, multi-agent chemotherapy for ALL should be started with the aim of achieving remission and improvement in full blood count prior to proceeding, preferably with vaginal delivery. Our case represents the suitability of treating pregnancy-associated ALL presenting during the second or early third trimester with the BFM 2000 for favorable outcomes.

## Conclusions

Our case report introduces a novel approach to tailoring the BFM 2000 induction chemotherapy protocol for pregnant patients with ALL, acknowledging the need for a cautious balance between effective cancer treatment and fetal protection. By replacing pegaspargase with safer alternatives and modifying the IT component, we successfully managed the disease, culminating in the safe delivery of a healthy baby. This innovative treatment strategy adds to the limited evidence in the field, potentially guiding future management decisions for pregnant patients with ALL.
